# Intermittent Levosimendan Administration for Advanced Heart Failure Treatment in Adults with Congenital Heart Disease (Levo-ACHD Study)

**DOI:** 10.3390/medicina62061170

**Published:** 2026-06-16

**Authors:** Flavia Fusco, Ippolita Altobelli, Vito Casale, Nunzia Borrelli, Giovanni Domenico Ciriello, Rosaria Barracano, Assunta Merola, Nicola Grimaldi, Michela Palma, Giovanni Papaccioli, Anna Correra, Diego Colonna, Giancarlo Scognamiglio, Berardo Sarubbi

**Affiliations:** Adult Congenital Heart Disease Unit, Monaldi Hospital, 80131 Naples, Italy

**Keywords:** heart failure, congenital heart disease, ACHD, levosimendan, inotropes

## Abstract

**Background and Objective:** Heart failure (HF) is a major cause of morbidity in adults with congenital heart disease (ACHD), who may also have limited access to transplant. Intermittent levosimendan administration has shown benefit in advanced HF due to acquired heart disease, but currently, there are no data on ACHD. We aimed to evaluate the effects of this treatment in ACHD patients with advanced heart failure, focusing on both clinical status and objective outcome measures. **Materials and Methods:** We conducted a single-center retrospective analysis of ACHD patients aged >18 years with advanced HF who received ≥ three intermittent levosimendan infusions (either 12.5 mg once monthly or 6.25 mg every two weeks over a 6 h infusion) between March 2020 and January 2025 at a tertiary ACHD center. Clinical outcomes during follow-up were compared with those in the year preceding treatment. Primary endpoints included safety and HF-related adverse events, particularly HF hospitalizations. Secondary endpoints included changes in New York Heart Association (NYHA) class, nt-pro-B-natriuretic peptide (nt-proBNP) values, and ventricular systolic function assessed by echocardiography. **Results:** Twelve patients (median age 44.6 years, 25% female) were included, with heterogeneous congenital diagnoses and advanced HF. Five patients had a systemic right ventricle (sRV) and one had a single ventricle with previous Fontan palliation. During a median follow-up of 1.3 years, intermittent levosimendan was well-tolerated, with no treatment-limiting adverse events. Two patients (16%) required hospitalization for HF during follow-up compared with 8 (66%) in the year preceding treatment. The incidence of HF hospitalizations decreased from 0.83 to 0.20 events per person-year during follow-up (*p* = 0.03), although findings should be interpreted cautiously given the small sample size and retrospective design. NYHA functional class improved significantly (*p* = 0.005). While no significant changes were observed in NT-proBNP or left ventricular ejection fraction, patients with a systemic right ventricle (sRV) showed an increase in right ventricular fractional area change (27 ± 7.4% to 30.6 ± 7%, *p* = 0.02); however, this observation should be regarded as exploratory given the limited sample size. Two deaths occurred, consistent with the severity of the underlying disease and not directly attributable to levosimendan and the Fontan patient received a successful heart and liver transplant. **Conclusions:** In a small, real-world cohort of ACHD and advanced HF, intermittent levosimendan administration was safe and associated with improved symptoms, reduced HF hospitalizations, and signals of enhanced systemic right ventricular function. These hypothesis-generating findings may help inform future multicenter studies in ACHD patients with advanced HF, suggesting a potential role for intermittent levosimendan in selected patients, while highlighting the need for prospective, adequately powered studies to confirm its efficacy and better define optimal patient selection.

## 1. Introduction

Heart failure (HF) is highly prevalent among adults with congenital heart disease (ACHD), and its treatment in this complex population presents unique challenges that differ substantially from acquired heart disease. Complex underlying anatomy, prior surgical repairs, heterogeneous hemodynamics, and frequent comorbidities complicate HF management in this population. Evidence for HF treatments is limited, as ACHD patients are often excluded from randomized trials, and large dedicated studies are lacking. Timing of referral for advanced therapies, including cardiac transplantation or mechanical circulatory support, is particularly difficult in ACHD. Many patients are deemed ineligible due to complex anatomy, prior surgical history, elevated immunologic sensitization, or end-organ dysfunction. This contributes to high waitlist morbidity and mortality in ACHD transplant candidates [1,2]. Conventional high-urgency listing strategies and hemodynamic optimization are used to mitigate risk, but bridging therapies are often needed. Mechanical circulatory support is often underutilized in ACHD, partly due to the lack of devices tailored to diverse anatomies and the technical challenges posed by complex physiologies (e.g., Fontan circulation) [3]. In patients with refractory HF, intermittent administration of levosimendan—a calcium-sensitizing inotrope with vasodilatory properties—has been reported to improve hemodynamic status and quality of life, reduce HF hospitalizations [4], facilitate optimization of background HF therapy and reduce waitlist morbidity [5]. Intermittent levosimendan infusions have been recommended as a viable option for bridge-to-cardiac transplantation in selected patients with acquired heart disease, according to the 2024 Guidelines of the International Society for Heart and Lung Transplantation [6]. However, in patients with congenital heart disease, evidence remains extremely limited. To date, data are largely confined to reports of potential benefit in the perioperative setting in pediatric populations [7,8] and to a single case series describing three adults with advanced disease [9]. ACHD patients have been largely excluded from major HF studies; therefore, it remains uncertain whether the efficacy and safety of levosimendan observed in patients with acquired heart disease can be directly extrapolated to this population, considering the different pathophysiology encompassing individuals with systemic right ventricle (sRV), Fontan physiology, or other complex anatomies. This underscores the need for population-specific evaluations.

Therefore, the present study aimed to describe the experience of our tertiary adult congenital heart disease center with intermittent levosimendan administration in ACHD patients with advanced heart failure, evaluating safety, HF outcomes, and potential bridging benefits.

## 2. Methods

### 2.1. Patient Selection and Data Collection

We retrospectively reviewed the digitally stored medical records at our tertiary center for all patients with a diagnosis of congenital heart disease aged >18 years with advanced HF who received at least three levosimendan infusions between March 2020 and January 2025. Advanced HF was defined according to contemporary guidelines [10] and was established by the treating ACHD heart failure team. Patients fulfilled one or more criteria consistent with advanced HF, including severe ventricular dysfunction despite optimized medical therapy, persistent NYHA class III–IV symptoms, recurrent heart failure hospitalizations, progressive clinical deterioration, elevated natriuretic peptide levels, impaired exercise capacity, worsening hemodynamics, or consideration for advanced heart failure therapies such as heart transplantation.

In all cases, the indication for intermittent levosimendan therapy was established during multidisciplinary assessment by congenital heart disease and heart failure specialists. The minimum of three infusions for study inclusion was intended to exclude patients who received levosimendan only during transient episodes of acute decompensation and who subsequently recovered, as these patients would not reflect the chronic advanced HF population targeted by this study. Exclusion criteria were absence of a congenital heart disease, age ≤18 years, receipt of fewer than three levosimendan infusions, perioperative only or emergency use of levosimendan, and non-HF indications (e.g., use of levosimendan exclusively before invasive hemodynamic assessment to evaluate potential transplant eligibility). Patients’ medical records were reviewed to collect data on previous medical history, previous surgeries, medical treatment and clinical status at the latest follow-up. Adverse events recorded during follow-up included any hospitalization for cardiac causes; acute heart failure, defined as the rapid onset or worsening of symptoms and signs of fluid overload requiring urgent medical evaluation and treatment; sustained arrhythmias, defined as abnormal heart rhythms persisting for >30 s or requiring immediate medical intervention (electrical cardioversion or pharmacological treatment) due to hemodynamic instability; appropriate implantable cardioverter/defibrillator (ICD) therapies (antitachycardia pacing or shocks); acute kidney injury (AKI), defined as a clinical syndrome characterized by a rapid decline in renal function occurring within hours or days; death; and the need for heart transplantation.

The study was conducted in accordance with the principles of the Declaration of Helsinki and was approved by the Institutional Ethics Committee (Comitato Etico Territoriale Campania 2, University of Campania “Luigi Vanvitelli”—Levo-ACHD; approval No. 23654, 7 August 2025). Given the retrospective nature of the study and the use of data collected during routine clinical care, the Institutional Review Board waived the requirement for written informed consent.

### 2.2. Levosimendan Administration Protocol

At our institution, the decision to initiate intermittent levosimendan administration was made following a multidisciplinary case review, with a shared agreement that no alternative therapeutic options were available for the patient.

Patients considered for intermittent levosimendan infusion could include those with advanced HF and the following:

- Heart failure with reduced ejection fraction (HFrEF; EF < 35%) refractory to guideline-directed medical therapy.
- HFrEF patients already listed for heart transplantation with ongoing clinical instability.
- HFrEF patients on optimized medical therapy with worsening symptoms and a New York Heart Association (NYHA) functional class >2, or recent HF hospitalization, prior to listing for transplantation, as a last attempt to achieve clinical recovery.
- Heart failure with preserved ejection fraction (HFpEF) unresponsive to conventional therapy.

**Figure 1:**
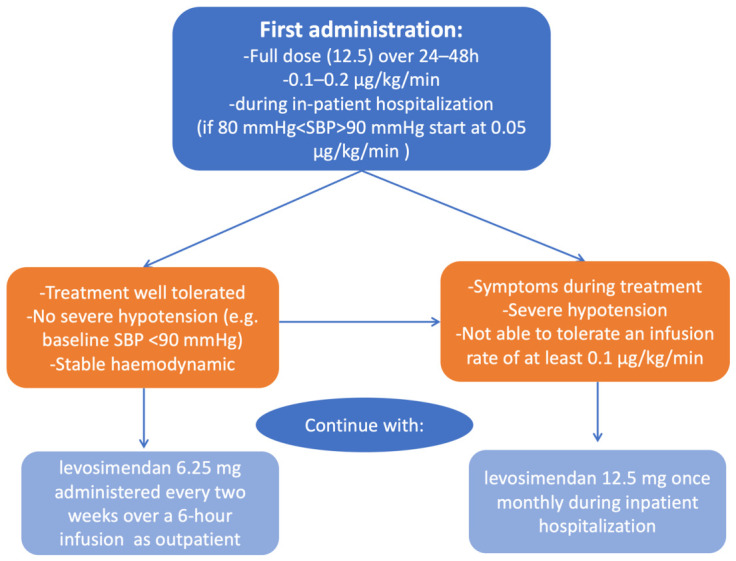
Levosimendan administration protocol. SBP = systolic blood pressure.

All patients evaluated for levosimendan administration at our institution undergo a comprehensive baseline assessment, including electrocardiography, physical examination, NYHA functional class evaluation, transthoracic echocardiography, and laboratory testing. Levosimendan was administered according to a standardized protocol with periodic infusions (Figure 1), which is based on the previous literature in patients with acquired heart disease [4]. The first administration is usually a full dose (12.5 mg) over a 24–48 h period and was performed in naïve patients during inpatient hospitalization to allow close monitoring for potential adverse events, particularly symptomatic hypotension and ventricular arrhythmias, as well as symptom evolution during infusion. Patients with a baseline systolic blood pressure (SBP) <80 mmHg are generally considered ineligible for levosimendan therapy. In patients with a baseline SBP between 80 and 90 mmHg, infusion is generally initiated at a reduced rate of 0.05 μg/kg/min. During infusion, patients undergo continuous ECG monitoring and periodic non-invasive blood pressure measurements. If SBP remains stable or within safety limits, and if there is no evidence of new ventricular arrhythmia at ECG monitoring, the infusion rate might be increased after the first 60 min, up to a maximum of 0.2 μg/kg/min. Patients who tolerate the initial infusion subsequently receive outpatient treatment, consisting of levosimendan 6.25 mg administered every two weeks over a 6 h infusion. More fragile patients, or those with significant hypotension who are unable to tolerate an infusion rate of at least 0.1 μg/kg/min, continue treatment with 12.5 mg once monthly during inpatient hospitalization.

### 2.3. Echocardiography

Comprehensive echocardiographic exams were periodically performed utilizing a GE Vivid-E80 machine (GE Healthcare, Wauwatosa, WI, USA). Standard M-mode, 2-dimensional, color, pulsed and continuous-wave Doppler images were acquired according to the European Association of Cardiovascular Imaging guidelines [11]. All measurements were obtained offline from stored images by a single experienced observer trained in ACHD echocardiography. Left ventricular (LV) EF was assessed with Simpson’s biplane method. For patients with an sRV, systolic function was assessed using a multiparametric approach with a combination of right ventricular (RV) systolic function parameters: TAPSE, S-wave, and fractional area change (FAC). Systolic pulmonary artery pressure (SPAP) was measured from the tricuspid regurgitation jet velocity, applying the modified Bernoulli equation [11], as recommended. Analogously, in patients with an sRV, ventricular pressure was estimated from the mitral regurgitation jet. Valvular regurgitation/stenosis severity was assessed using multiparametric evaluation, in agreement with guidelines [11]. Right atrial pressure was derived from the inferior vena cava diameter and collapsibility [11].

### 2.4. Endpoints

Primary endpoints included safety of levosimendan treatment and efficacy in reducing the number of adverse events in ACHD patients. Events at follow-up were compared to those occurring in the year before treatment initiation. Primary efficacy endpoints included the following:

(a) Death for any reason;
(b) New transplant listing/necessity of mechanical circulatory support (MCS);
(c) Unscheduled hospital admission for HFl;
(d) Occurrence of sustained ventricular tachycardia (VT), defined as VT > 30 s or non-sustained VT with symptoms, or ICD appropriate therapies;
(e) A composite of all the previous occurrences.

All patients were evaluated according to the overall treatment period during which intermittent levosimendan therapy was administered, irrespective of the setting of infusion.

Safety was defined as the absence of relevant adverse events potentially related to levosimendan administration, such as symptomatic hypotension requiring intervention, clinically significant arrhythmias, or treatment discontinuation. Minor events not requiring intervention were not systematically collected in this retrospective analysis. Both efficacy and safety events were adjudicated by a consensus of the investigators.

Secondary endpoints were quantifiable parameters, including nt-pro-B-natriuretic peptide (nt-proBNP) and LV and sRV systolic function, assessed by echocardiography. One additional endpoint was improvement in the New York Heart Association (NYHA) class.

### 2.5. Statistical Analysis

Statistical analysis was performed using R (version 4.0.5). The Shapiro–Wilk test was used to assess data normality. Continuous variables were reported as mean ± SD or median [IQR], according to data distribution. Comparisons between baseline and follow-up data were assessed with the t-test or Wilcoxon’s rank-sum test for paired samples. Categorical variables were presented as frequencies (percentage of total). Differences in proportions were evaluated with McNemar’s test. Incidence rates were calculated as events per person-year and compared between the pre-treatment period and follow-up using differences in incidence rates. *p*-values < 0.05 were considered statistically significant.

## 3. Results

### 3.1. Study Population

Twelve patients met the inclusion criteria: there were three (25%) females, with median age of 44.6 [35–61.3] years. Demographic data, diagnosis and baseline characteristics of the study population are summarized in Table 1. Half the patients had a biventricular physiology with an LV sustaining systemic circulation (six patients = 50%), while a systemic right ventricle (sRV), was present in five (41%) patients (four due to congenitally corrected transposition of the great arteries and one due to transposition of the great arteries following atrial switch repair), and one patient had an univentricular heart with LV morphology and received Fontan palliation with an extracardiac conduit. Half the patients had severe disease complexity according to the Anatomical Classification [12]. Six patients had at least two surgical and/or percutaneous procedures and four (33%) had an implantable defibrillator. Associated defects, additional details on previous surgical/percutaneous procedures and residual defects are presented in Supplemental Table S1. All patients had a history of at least one HF-related hospitalization, and 50% had multiple previous acute HF decompensation episodes. At least one HF hospitalization was reported in eight patients (66%) in the year preceding treatment. All patients had symptoms, as demonstrated by an NYHA class ≥ two. Median nt-proBNP was 1576 [706–3023] pg/mL. Impaired exercise capacity was demonstrated by a median distance of 270 [245–366] meters walked in 6 min. A baseline cardiopulmonary test was available in only one patient and it confirmed severely reduced exercise capacity with a peakVO_2_ of 8 mL/kg/min. End-organ damage consistent with renal impairment was present in three (25%) patients and liver dysfunction with subsequent hepatocellular carcinoma was present in one patient. The Fontan-palliated patient was already on the heart and live transplant list at baseline and the other two patients were evaluated for possible future listing. Baseline EF among patients with an sLV (including the single-ventricle patient) was 37.6 ± 8%, while baseline FAC for patients with an sRV was 27 ± 7.4%. However, four (66%) out of the six patients with sLV had biventricular failure, with three showing predominant RV failure (FAC of 25 ± 11%). Median systolic pulmonary pressure estimated by echocardiography in patients with sLV was 38 [29.5–62] mmHg. Cardiac catheterization data were available for five (42%) patients (four with an sLV and one with sRV) and are presented in Table 2. Three patients showed pulmonary arterial hypertension: one following atrial switch procedure, one with previous atrial septal defect repair with a fenestrated device and one with previous tetralogy of Fallot repair. Before starting levosimendan, all patients were receiving maximally tolerated HF guideline-directed medical therapy, optimized according to contemporary recommendations [10] and individual clinical status. Baseline medications are summarized in Table 1.

### 3.2. Levosimendan Treatment

The indication for treatment initiation was acute decompensation in four cases (33%), and chronic HF in the other cases, with one already on the transplant list at baseline. After the first in-hospital administration, only three patients (25%) were selected for outpatient treatment. However, one patient with single-ventricle physiology and a previous Fontan operation did not tolerate outpatient administration because of transient severe hypotension occurring two hours after infusion initiation; therefore, subsequent cycles were scheduled as in-hospital treatments at one-month intervals. All other patients underwent monthly in-hospital administration of 12.5 mg.

No other severe adverse events requiring treatment interruption occurred during levosimendan infusions, and no episodes of sustained ventricular tachycardia were recorded. Two patients temporarily discontinued treatment: one due to patient preference related to difficulty in reaching the hospital, although this patient subsequently experienced clinical deterioration and treatment was reinitiated; the second patient initially showed clinical improvement, leading to a planned treatment interruption, but developed fluid overload two months after suspension, prompting reinitiation of the therapy. One patient continued treatment at a local hospital after the first seven doses were administered.

### 3.3. Follow-Up and Secondary Endpoints

**Figure 2:**
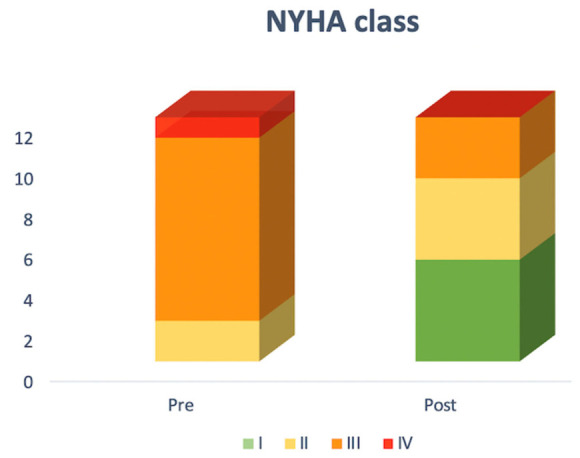
NYHA class change during follow-up.

Median follow-up after initiation was 1.3 [0.7–2.3] years. Overall, patients received a median of 8.5 [6.2–15] doses over a median of 10.7 [5.8–16.7] months. The main findings at the last evaluation and comparison with baseline data are shown in Table 3. During follow-up, HF therapy was optimized, despite no significant change in the proportion of patients receiving each class of medication. In particular, the proportion of patients receiving ARNI and SGLT2 inhibitors increased from 7 to 10 and from 8 to 11, respectively. While the number of patients treated with beta-blockers remained stable, the mean bisoprolol dose increased from 3.3 ± 0.8 mg/day at baseline to 4.5 ± 1.0 mg/day at the last follow-up, reflecting further optimization of background heart failure therapy. No significant changes in pulmonary vasodilator prescriptions were observed during follow-up. At the last evaluation, there was no significant improvement in ntproBNP values. However, patients reported a significant reduction in symptoms (Figure 2). Moreover, blood tests did not demonstrate significant renal function changes. Patients with sLV did not demonstrate a significant increase in EF. Nevertheless, patients with an sRV showed a significant improvement in FAC. However, it should be highlighted that, given the small sample size, subgroup analyses should be interpreted cautiously and regarded as exploratory, requiring confirmation in larger studies.

### 3.4. Outcome

During follow-up, two deaths occurred: one patient with pulmonary hypertension died from an intercurrent pulmonary infection, and one patient experienced an arrhythmic death during hospitalization for routine levosimendan administration, occurring the day after treatment. The patient with arrhythmic death had an sRV with biventricular failure and a baseline history of previous VT for which they had already undergone a subcutaneous ICD implantation for secondary prevention. No other ventricular tachycardia occurred. Three HF hospitalizations occurred in two patients: one with previous atrial septal defect closure and pulmonary hypertension and one with an sRV and biventricular failure. The latter experienced two acute HF episodes, in one case accompanied by acute kidney injury, which was attributed to hemodynamic compromise and which recovered completely. Both patients who had HF at follow-up were managed with i.v. diuretics and optimized medical therapy. The Fontan patient already listed at baseline received a successful heart and liver transplant at another institution. Two patients are currently under evaluation for transplant list inclusion after hemodynamic improvement following levosimendan (including the patient with sRV and biventricular failure mentioned above) and are still undergoing intermittent levosimendan treatment as a bridge to transplant. HF admission incidence decreased from 0.83 (95% CI 0.39–1.50) events/person-year before treatment to 0.20 (95% CI 0.04–0.60) during follow-up (incidence rate difference, *p* = 0.03). Outcome data are reported in Table 4.

## 4. Discussion

In this real-world cohort of complex ACHD patients with advanced HF, intermittent levosimendan administration was safe and associated with significant symptom improvement, increased RV FAC in the small subgroup of patients with an sRV, and a reduction in HF admissions during follow-up compared with the year prior to treatment initiation. Although the changes in NT-proBNP and LVEF did not reach statistical significance, a numerical improvement was observed during follow-up. Given the small sample size of this exploratory cohort, these findings should be interpreted cautiously but may still suggest a potentially clinically meaningful signal. Moreover, considering the limited sample size, more complex approaches, such as time-varying adjustment models to assess event incidence, were not considered sufficiently robust. Therefore, the results should be interpreted as exploratory and hypothesis-generating. The observed improvement in sRV function should be hypothesis-generating only, given that this analysis was limited to a small subgroup of patients. Although arrhythmic death occurred in a patient with end-stage disease and severe ventricular dysfunction, a potential contributory role of levosimendan cannot be completely excluded. This event highlights the need for careful prospective safety monitoring in future studies. In a single-ventricle patient with Fontan circulation, levosimendan was successfully used as a bridge to transplant. The reduction in HF events is clinically meaningful, as recurrent hospitalizations substantially contribute to morbidity, healthcare utilization, and reduced quality of life in ACHD patients [12,13].

Adult survival in complex congenital heart disease has improved substantially, leading to a growing population with late-onset HF that often remains refractory to conventional therapies. Standard HF treatments, validated in acquired HF, have limited efficacy in ACHD, particularly in patients with Fontan physiology [14,15] or sRV [16,17], despite recent promising results from novel therapies [18,19,20,21]. In our cohort, a statistically significant improvement was observed only in FAC among patients with sRV, though the small sample size may have limited the ability to detect additional significant benefits. Favorable but non-significant trends were noted for NT-proBNP reduction and LV EF improvement.

Mechanistically, levosimendan enhances cardiac performance by increasing contractility without raising myocardial oxygen demand and by reducing afterload through vasodilation, mediated by calcium sensitization and Ca^2+^-dependent opening of ATP-sensitive K^+^ channels in vascular smooth muscle cells [22]. These effects may be particularly beneficial in ACHD patients with failing sRV or longstanding pressure-overload conditions, where traditional inotropes can exacerbate arrhythmogenic risk and adverse remodeling. The prolonged action of levosimendan’s active metabolite allows intermittent outpatient administration, potentially sustaining clinical benefits between treatments. While intermittent levosimendan use is well documented in general advanced HF populations, evidence specific to ACHD remains limited. A recent case series of three ACHD patients with advanced HF demonstrated symptomatic improvement and avoidance of hospital admissions with pulsed levosimendan, supporting its potential role in clinical practice [9]. Other studies have shown levosimendan’s efficacy in treating postoperative low cardiac output syndrome (LCOS) [23] and in reducing LCOS incidence when administered prophylactically in children with congenital heart disease [7,8].

In broader HF populations, levosimendan’s unique pharmacologic properties have justified repeated intermittent use. The LION-HEART study, a double-blind, randomized, placebo-controlled trial, demonstrated reductions in NT-proBNP values and HF hospitalizations with intermittent levosimendan in outpatients with advanced chronic HF [24], and similar findings were identified in a subsequent trial [25]. These data support the Class 2A recommendation for intermittent levosimendan in patients with organ hypoperfusion awaiting transplant, according to the 2024 International Society for Heart and Lung Transplantation Guidelines [6]. Our experience suggests the potential role for levosimendan as a bridge-to-transplant therapy in complex ACHD populations. This may represent a significant advantage in the setting of refractory heart failure and limited donor availability, as often occurs in ACHD. In our cohort, the two patients currently considered for transplant achieved hemodynamic stabilization with levosimendan. Further studies are warranted to determine whether intermittent levosimendan can reduce morbidity and mortality while on the transplant waitlist for ACHD patients. A recent multicenter retrospective study also showed reduced HF admissions with intermittent levosimendan in patients not eligible for transplant, supporting its use as a destination therapy [26]. However, a randomized trial in patients with severe, potentially reversible cardiogenic shock on extracorporeal membrane oxygenation (ECMO) found that early levosimendan did not significantly shorten time to ECMO weaning compared with the placebo [27]. In addition, a randomized controlled study in a small cohort of patients with HF with preserved ejection fraction (HFpEF) showed that pulsed levosimendan administration was associated with reductions in pulmonary capillary wedge pressure and improvements in exercise tolerance [28]. These preliminary findings have stimulated further investigation into HFpEF associated with pulmonary hypertension, including the development of an oral formulation of levosimendan [29]. On this basis and given the high prevalence of refractory HFpEF in ACHD, at our institution, we also started to consider levosimendan infusion in selected ACHD patients with preserved ventricular systolic function. Despite the fact that HFpEF is frequently described in patients with repaired aortic coarctation [30], it is worth noting that two patients with this diagnosis included in our cohort had HF with severely reduced EF. Notably, a meta-analysis of eight randomized studies demonstrated that levosimendan improves right ventricular systolic function and reduces pulmonary artery pressure [31], which are findings of particular relevance to the ACHD population, encompassing a high proportion of RV failure.

Although arrhythmias are recognized among the potential adverse effects of levosimendan, the drug is generally considered to have a favorable safety profile compared with conventional inotropes, and ventricular arrhythmias appear uncommon due to its mechanism; it does not increase intracellular calcium or myocardial oxygen demand [4]. In studies involving congenital heart disease populations—mainly in pediatric and perioperative settings—hypotension and arrhythmias have been reported as the most frequent adverse events, while available evidence does not suggest increased mortality attributable to levosimendan [32]. Given the high prevalence of ventricular arrhythmias in adults with congenital heart disease due to surgical scars, myocardial fibrosis, and systemic ventricular dysfunction, the event observed in our cohort likely reflects the advanced stage of the underlying disease rather than a direct drug-related effect, although careful rhythm monitoring remains essential when intermittent levosimendan therapy is used in this population.

Unlike large trials in acquired HF, ACHD studies have largely been small cohorts or case series, limiting conclusions regarding the benefit magnitude and optimal dosing. Our study has several limitations. First, its retrospective, non-controlled design and small sample size preclude definitive causal inference and increase selection bias risk. Second, the heterogeneity of congenital lesions and physiology complicates generalizability, as responses may differ between subgroups (e.g., sRV vs. sLV vs. single-ventricle physiology). For this reason, we analyzed LV EF and sRV FAC separately, further reducing the sample size and statistical power. Therefore, given the small sample size, subgroup comparisons should be interpreted as exploratory analyses and considered hypothesis-generating only, warranting further investigation in larger cohorts. Third, objective measures such as exercise capacity, biomarker trends, and formal quality-of-life instruments were not systematically collected, emphasizing the need for prospective evaluation. Furthermore, given the retrospective design and concomitant optimization of background therapy during follow-up, the independent contribution of levosimendan and HF therapy cannot be established. The inclusion criterion requiring at least three levosimendan infusions, aimed at evaluating the effects of intermittent therapy on chronic HF outcomes, may have introduced survivorship bias by excluding patients who deteriorated early and potentially overestimating both tolerability and efficacy. Finally, although levosimendan was generally well tolerated, rare adverse events may not be captured in small cohorts. Nevertheless, despite all these limitations, we believe that our real-world single-center experience may provide preliminary data that could support the design of larger multicenter studies, which will be necessary to determine whether intermittent levosimendan therapy can help stabilize HF in ACHD patients and potentially serve as a bridge to heart transplantation.

## 5. Conclusions

In this real-world cohort of ACHD and advanced HF, intermittent levosimendan was feasible and associated with symptomatic improvement, fewer HF hospitalizations and a signal toward improved systemic right ventricular function was observed, although the latter finding is exploratory. Given the retrospective design, small sample size, and concomitant optimization of background heart failure therapy in this study, these results should be considered hypothesis-generating. Prospective multicenter studies are needed to confirm these observations and better define the role of intermittent levosimendan in ACHD.

## Figures and Tables

**Table 1:** Demographic data and baseline characteristics in the study population (N = 12).

Sex (female)	3 (25%)
Age (years)	44.6 [35–61.3]
Weight (kg)/Height (cm)	71 ± 17/167 ± 9
Main diagnosis	
Aortic Coarctation	2
Atrial septal defect	2
PAPVD	1
Tetralogy of Fallot	1
AVSD with straddling valve	1
TGA with atrial switch repair	1
ccTGA	4
Systemic ventricle morphology	
Biventricular physiology with sLV	6 (50%)
Biventricular physiology with sRV	5 (41%)
Univentricular heart with sLV after Fontan palliation with an extracardiac conduit	1 (8%)
Genetic disorder	
Turner syndrome	1
Comorbidities	
Hepatocellular carcinoma	1 (8%)
CKD	2 (17%)
COPD	4 (33%)
Hypothyroidism	2 (17%)
Renal impairment	3 (25%)
Cardiovascular risk factors	
Diabetes	1 (8%)
Hypertension	2 (17%)
Obesity	3 (25%)
Dyslipidemia	3 (25%)
Chronic coronary syndrome and previous PTCA	2 (17%)
PM/ICD	1 (8%)/4 (33%)
NYHA class (II/III/IV)	2 (17%)/9 (75%)/1 (8%)
Previous hospitalization for HF	12 (100%)
HF hospitalization > 1	6 (50%)
History of SVT	4 (33%)
History of VT	5 (42%)
Previous heart transplant referral	3 (25%)
Baseline rhythm	
Sinus	8 (66%)
Ventricular pacing	2 (17%)
Atrial fibrillation	2 (17%)
Baseline SBP (mmHg)	108 ± 10
Baseline HR (bpm)	76 ± 12
Treatment	
ARNI	7 (58%)
Gliflozins	8 (66%)
ACEi/ARB	3 (25%)
Beta-blockers	9 (75%)
MRA	7 (58%)
Diuretics	8 (66%)
Pulmonary vasodilators	1 (17%)
Ivabradine	2 (17%)
DOAC	(25%)
Warfarin	2 (17%)
Antiplatelets	2 (17%)
Amiodaron	2 (17%)

Abbreviations: ACEis = angiotensin-converting enzyme inhibitors; ARBs = angiotensin-receptor blockers; AVSD = atrioventricular septal defect; ccTGAs = congenitally corrected transposition of the great arteries; CKD = chronic kidney disease; COPD = chronic obstructive pulmonary disease; DOACs = direct oral anticoagulants; HF = heart failure; HR = heart rate; ICD = implantable cardioverter/defibrillator; NYHA = New York Heart Association; PAPVD = partial anomalous pulmonary venous drainage; PM = pacemaker; PTCA = percutaneous transluminal coronary angioplasty; SBP = systolic blood pressure; SVT = supraventricular tachycardia; TGAs = transposition of the great arteries; sLV = systemic left ventricle; sRV = systemic right ventricle; VT = ventricular tachycardia. Continuous variables are expressed as mean ± SD or median [IQR], according to data distribution.

**Table 2:** Baseline cardiac catheterization data (N = 5).

PAPm (mmHg)	26 ± 9
PCWP (mmHg)	14 ± 5
PVRi (Wu)	3 ± 1.9
CI (l/min/m^2^)	3 ± 1.8

Abbreviations: CI = cardiac index; PAPm = mean pulmonary artery pressure; PCWP = pulmonary capillary wedge pressure; PVRi = indexed pulmonary vascular resistance.

**Table 3:** Comparison between baseline and follow-up data.

	Baseline	Follow-Up	*p*-Value
NtproBNP (pg/mL)	1576 [705–3023]	967 [241–2263]	0.3
Creatinine (mg/dL)	0.84 ± 0.25	0.88 ± 0.17	0.4
NYHA class III–IV	10 (83%)	3 (25%)	0.005
LV EF * (%)	37.6 ± 8	43.6 ± 5	0.1
RV FAC ** (%)	27 ± 7.4	30.6 ± 7	0.02
HF treatment			
ACEi/ARB	3 (25%)	1 (8%)	0.3
ARNI	7 (58%)	10 (83%)	0.2
SGLT2i	8 (66%)	11 (92%)	0.1
BB	9 (75%)	9 (75%)	1
MRA	7 (58%)	8 (67%)	0.6
Diuretics	8 (66%)	9 (75%)	0.6

**Abbreviations:** ACEis = angiotensin-converting enzyme inhibitors; ARBs = angiotensin-receptor blockers; BBs = beta-blockers; EF = ejection fraction; FAC = fractional area change; HF = heart failure; LV = left ventricle; MRAs = mineral corticoid receptor antagonists; NYHA = New York Heart Association; RV = right ventricle; SGLT2is = sodium–glucose cotransporter 2 inhibitors; * = only patients with systemic left ventricle; ** = only patients with systemic right ventricle.

**Table 4:** Outcome in the study population (N = 12).

Death	2 (17%)
HF hospitalization	2 (17%)
AKI	1 (8%)
Transplant	1 (8%)
New consideration for transplant	2 (17%)
VT	1 (8%)

**Abbreviations**: AKI = acute kidney injury; HF = heart failure; VT = ventricular tachycardia.

## Data Availability

The data presented in this study are available on request from the corresponding author due to privacy reasons.

## Supplementary Materials

The following supporting information can be downloaded at: https://www.mdpi.com/article/10.3390/medicina62061170/s1, Supplemental Table S1. Associated defects and details on previous surgical/percutaneous procedures and residual defects.
