# Supplementary material for: Intermittent Levosimendan Administration for Advanced Heart Failure Treatment in Adults with Congenital Heart Disease (Levo-ACHD Study)
Source: Medicina (Kaunas). 2026 Jun 16;62(6):1170. doi: 10.3390/medicina62061170 (PMC13303742; doi:10.3390/medicina62061170)
Supplement: Supplementary file 1 [file medicina-62-01170-s001.zip › medicina-4329317-supplementary.pdf]

Supplemental Table S1. Associated defects and details on previous surgical/percutaneous procedures and residual defects

Associated defects	1 Atrial septal defect<br>1 Bicuspid aortic valve<br>1 Right isomerism<br>2 Ventricular septal defects<br>1 Pulmonary stenosis<br>1 Coarctation repair
Surgical/percutaneous procedures	2 Coarctation repair (2 end to end anastomosis, 1 subclavian flap)<br>3 Atrial septal defect closure (1 with a fenestrated device, 2 surgically)<br>1 Glenn and Fontan procedure<br>1 Tetralogy of Fallot repair<br>1 Tricuspid valve annuloplasty and mitral valve edge-to-edge repair<br>1 Correction of anomalous pulmonary veins drainage<br>1 Percutaneous closure of left atrial appendage<br>1 Mustard operation<br>2 ventricular septal defect closure<br>1 Pulmonary stenosis relief<br>3 Blalock Taussing shunt<br>1 Pulmonary artery banding<br>1 Rashkind balloon atrial septostomy<br>1 Fontan conduit stenting<br>1 Fontan conduit replacement
Total number of procedures	
0	2(17%)
1	4(33%)
2	5(42%)
5	1(8%)
Residual defects	2 Pulmonary arterial hypertension<br>1 Severe aortic stenosis<br>5 Severe tricuspid regurgitation<br>1 Severe pulmonary regurgitation<br>1 Mitral regurgitation
